# BikDDA, a Mutant of Bik with Longer Half-Life Expression Protein, Can Be a Novel Therapeutic Gene for Triple-Negative Breast Cancer

**DOI:** 10.1371/journal.pone.0092172

**Published:** 2014-03-17

**Authors:** Shiping Jiao, Minqing Wu, Feng Ye, Hailin Tang, Xinhua Xie, Xiaoming Xie

**Affiliations:** 1 Department of Breast Oncology, Sun Yat-Sen University Cancer Center, Guangzhou, Guangdong, China; 2 Sun Yat-Sen University Cancer Center, State Key Laboratory of Oncology in South China, Collaborative Innovation Center for Cancer Medicine, Guangzhou, Guangdong, China; 3 Department of Cancer Prevention Center, Sun Yat-Sen University Cancer Center, Guangzhou, Guangdong, China; 4 Zhongshan School of Medicine, Sun Yat-Sen University, Guangzhou, Guangdong, China; Lovelace Respiratory Research Institute, United States of America

## Abstract

Our previous studies showed that BikDD, a constitutively active mutant form of Bik, exhibited powerful antitumor effects in preclinical pancreatic, lung and breast cancer models. Howerver, the antitumor activity of BikDD in triple-negative breast cancer (TNBC) is unknown. Here we show that aberrant expression of p-ERK1/2 was a meaningful molecular phenotype in TNBC patients, and can be an obstacle for treatment because of the converse correlation with Bik. A novel mutant, BikDDA, in which Ser^124^ was changed to Alanine to block BikDD phosphorylation by p-ERK1/2 prevented subsequent ubiquitin-proteasome degradation. BikDDA showed a prolonged half-life and enhanced pro-apoptotic ability in TNBC cells compared with BikDD. Moreover, aberrant expression of p-ERK1/2 was associated with 5-fluorouracil resistance in breast cancer patients and BikDDA enhanced the therapeutic effects of 5-fluorouracil in vitro.

## Introduction

Breast cancer is a heterogeneous disease and its incidence is increasing worldwide [1]. Luckily, with the development of targeted drugs, the outcome of some subgroups of breast cancer was improved. However, 15–20% of breast cancer, defined as triple-negative breast cancer (TNBC) by absence of estrogen receptor, progesterone receptor and Her2 receptor, did not benefit from targeted drugs because of resistance to existing hormonal or Her2 targeted therapy [2], [3]. TNBC has a high rate of relapse and the worst prognosis among all breast cancers.

The pathways that drive proliferation of TNBC remain somewhat elusive. But aberrant activation of the Ras-Raf-MEK-ERK pathway in TNBC has already been noticed [4]-[6]. This signaling cascade plays a pivotal role in the signal transduction from growth factor receptors to transcription factors and was frequently found constitutively activated in solid tumors [7]. ERK1/2 is a central downstream effector of the Ras-Raf-MEK-ERK pathway. Phosphorylated (P) -ERK1/2, the active form of ERK1/2, regulates gene expression to promote proliferation and differentiation and prevents apoptosis by directly phosphorylating apoptotic regulators of Bcl-2 family [7], [8].

The Bcl-2 family of proteins play a vital role in the intrinsic pathway of apoptosis and can be divided into three subgroups according to Bcl-2 homology(BH) domains : multidomain pro-apoptotic(BAX,BAK), BH3-only pro-apoptotic (BID,BIM,BAD,BIK,NOXA,PUMA,BMF,HRK) and multidomain anti-apoptotic (BCL-2,BCL-XL,BCL-W,MCL-1,BFL-1/A1). The balance among pro-apoptotic and anti-apoptotic Bcl-2 family members determines the outcome of apoptosis signals [9]. Overexpression of anti-apoptotic members but lack of pro-apoptotic can be an important molecular mechanism for evasion of apoptosis [10]–[13]. So antagonizing anti-apoptotic members of Bcl-2 family to promote apoptosis is a good strategy for targeted anticancer drug discovery [14]–[16].

Bik, as a BH-only pro-apoptotic member, binds with Bcl-2, Bcl-Xl or Mcl-1 to replace BAX or BAK, which forms BAX/BAK oligomerization and then triggers mitochondrial outer membrane permeabilization, cytochrome c into cytoplasm, caspase-9 activation and at last cell apoptosis [9]. For its greatest binding affinity and broadest binding pattern with Bcl-2 homology partners, we selected Bik as a cancer targeted therapeutic gene [17]. To get a more potent gene for cancer therapy, we have previously mutated the residues threonine 33 and serine 35 of Bik to aspartic acid to create the constitutively phosphorylated form, BikDD, with enhanced binding affinity to anti-apoptotic members Bcl-2, Bcl-Xl or Mcl-1 and greater pro-apoptotic activity [18]. With targeted and efficient expression harnessed by tissue-cancer-specific promoter and VP16-GAL4-WPRE integrated systemic amplifier, BikDD as a therapeutic gene successfully eradicated pancreatic, lung and breast tumors in preclinical cancer models with virtually no toxicity [15], [16], [19], [20]. Moreover, the clinical trials of BikDD as a therapeutic gene for patients with advanced pancreatic cancer have reached to phaseI.

However, as an aggressive and intractable subtype of breast cancer, TNBC has the intrinsic resistance to apoptosis, but the molecular characteristics are still poorly understood. So it is not clear whether BikDD can eliminate TNBC cells efficiently.

In this study we (I) found aberrant expression of p-ERK1/2 was associated with a poor prognosis and was a molecular phenotype of TNBC; (II) investigated the relationship between p-ERK1/2 and Bik, Bcl-2, Bcl-Xl or Mcl-1; (III) generated a novel mutant therapeutic gene, BikDDA, according to the highly activated ERK1/2 molecular characteristic in TNBC; (IV) compared the half-life of BikDDA with that of BikDD and identified whether BikDDA exhibited a greater pro-apoptotic activity in TNBC cells; (V) explored whether higher expression of p-ERK1/2 contributed to 5-fluorouracil (5-FU) resistance and whether BikDDA enhanced the therapeutic effects of 5-FU or not.

## Materials and Methods

### Patients and samples

Samples used in immunohistochemistry were collected from 113 female breast cancer patients who were diagnosed by histo-pathology in Sun Yat-Sen University Cancer Center from October 2001 to September 2006. The end of observing was November 30, 2011. Specimens were formalin-fixed and embedded in paraffin by standard methodology after obtained during surgery. Eight samples used in western blotting analysis were obtained during surgery from eight female breast cancer patients (five TNBC and three NTNBC) who were diagnosed from June to November 2013. The whole group of patients did not receive any chemotherapy and radiation therapy before, and their complete clinico-pathological data, including age, histological type, lymph nodes status, tumor size, stage, local relapse, distant metastatic relapse, estrogen receptor status, progesterone receptor status and HER-2 status, were available and reviewed. Histological type, reclassified according to the WHO classification and stage of tumor, was based on the TNM staging system (American Joint Committee on Cancer classification).

This study was approved by the Ethics Committee of the Sun Yat-Sen University Cancer Center. Written informed consent was obtained from all study participants. The collection and use of patients’ tissues followed the procedures that are in accordance with the ethical standards as formulated in the Helsinki Declaration.

### Tissue microarray construction

The method of tissue microarray (TMA) construction was mentioned in our previous study [21].

### Immunohistochemical (IHC) staining and scoring system

The Labeled StreptAvidin Biotin Method was used in our study. We performed the IHC as the protocol (KIT-9710, Maxin FuZhou, China). Use the following antibodies: Bik, Bcl-2, Bcl-Xl, Mcl-1 (sc-10770, sc-130308, sc-8392, sc-56427; Santa Cruz, CA, USA) and pospho-ERK1/2(#9106, Cell signaling). Immunohistochemical staining was graded for intensity (0-negative, 1-weak, 2-moderate, and 3-strong; Fig S1) and percentage of positive cells [0, 1 (1–24%), 2 (25–49%), 3 (50–74%), and 4 (75–100%)] with discrepancies resolved by consensus. The grades were multiplied to determine a score. In this study, we characterized a low score (0–3) as low-expression and a high (≥ 4) as high-expression respectively. Assessments of the staining were scored in a double-blinded manner by two experienced pathologists.

### Cell lines and culture

MDA-MB-213, MDA-MB-435 and HEK 293 cell lines were obtained from American Type Culture Collection (Invitrogen, CA, USA), and freshly recovered from liquid nitrogen (<3 months) and were cultured at 37°C in an atmosphere of 5% CO_2_ in RPMI-1640 medium (Invitrogen, CA, USA) supplemented with 10% fetal bovine serum (FBS, GIBCO, Cappinas, Brazil). Antibiotic-Antimycotic (penicillin, streptomycin, and amphothericin B) were added according to the supplier’s instructions. All transfections were conducted using Lipofectamine 2000 (Invitrogen, CA, USA).

### Plasmid Construction and Site-directed Mutagenesis

PUK21 and pUK21-BikDD plasmids were stored by our lab. Site-directed mutagenesis was performed according to the manufacturer’s protocol (QuikChange Lightning Multi Site-Directed Mutagenesis Kit; agilent technologies). Serine 124 of BikDD was changed to Alanine acid by using the following primers: 5′- AACATAATGAGGTTCTGGAGAGCCCCGAACCCC-3′ and 5′-GGGGTTCGGGGCTCTCCAGAACCTCATTATGTT-3′. The sequences of BikDDA mutant constructs were confirmed by automated sequencing.

### Apoptosis assay

Annexin V/propidium iodide (PI) staining was performed for the detection of apoptotic cells using the Annexin V/Dead Cell Apoptosis Kit (Invitrogen, CA, USA) according to the manufacturer’s guidelines. Briefly, 5×10^5^ cells were transfected with 6ug of pUK21-BikDD or other agents. 28h after tranfection, the apoptotic cells were assessed by flow cytometric detection after being stained with Annexin V/propidium iodide.

### Cell proliferation assays

In a 24-well plate, 1.2×10^5^ cells were divided into each well. After attached, cells were transfected with pUK21-BikDD or other agents. 4 h later, 25 μg/ml 5-fluorouracil was added or not. Incubate 24h. MTT assay was performed as described in our previous study [22]. Cell counting was performed following the manufacturer’s guidelines (cell counting kit-8, dojindo, Japan).

### Western blotting analysis and pharmacological inhibitors

293 and 231 cells were transfected with same amount of pUK21-BikDD or pUK21-BikDDA. 16 hours later, cells were treated with pharmacological inhibitors as indicated and the harvested cells were lysed. Cell lysates were subjected to SDS–PAGE and protein bands were transferred to a polyvinylidene difluoride membrane (PVDF, Millipore, MA, USA). Primary Antibodies against Bik (sc-10770, Santa Cruz, CA, USA), β-actin (sc-47778, Santa Cruz, CA, USA), pospho-ERK1/2(#9106, Cell signaling technology, USA) and ERK1/2(#9102, Cell signaling technology, USA) and HRP-conjugated secondary antibodies (ab6721, ab6728; Abcam) were used. The immunoreactive bands were visualized with a chemiluminescent substrate (Santa Cruz, CA, USA). The relative protein expressions were analyzed by Gel pro software.

Pharmacological inhibitors: UO126 (5 μM, Beyotime, China), FR180204 (0.6 μM, Sigma, St. Louis, MO, USA), MG132 (10 μM, Beyotime, China) and cycloheximide (1 mM, Sigma, St. Louis, MO, USA) were utilized.

### Statistical analysis

Statistical analyses were performed with SPSS software, version 13.0 (SPSSInc., USA). Student’s t test, one-way ANOVA, Pearson’s chi-square test and Fisher’s exact test were used depending on the nature of the data. Overall survival was calculated from diagnosis to the date of death for any cause, and patients who were alive were censored at date of last follow-up visit. Disease-free survival was defined as time from diagnosis to disease progression or death, no matter which occurred first. Cumulative survival time and univariate analysis were calculated by the Kaplan–Meier method and the log-rank test, respectively. Multivariate analysis employed the Cox multivariate proportional hazard regression model. Each experiment was performed at least in triplicate. All p-values were two-tailed and p< 0.05 was considered to be statistically significant. Data are mean ± SD. * p<0.05, ** p<0.01, *** p<0.001.

## Results

### P-ERK1/2 was particularly over expressed in triple-negative breast cancer and predicted poor prognosis in breast cancer patients

Firstly we examined the correlation of p-ERK1/2 expression with clinicopathologic parameters. We found high levels of p-ERK1/2 were strikingly associated with distant metastasis, which implied p-ERK1/2 contributed to the clinical aggressiveness of breast cancer. What’s more, although p-ERK1/2 is always highly expressed, we did found triple-negative breast cancer showed much greater expression than non-triple negative breast cancer (38.6% vs 0.9%; p<0.001; Table 1). In keeping with this, higher p-ERK1/2 levels correlated with low expression levels of estrogen, progesterone and Her2 receptors respectively (Table 1).

**Table 1:** Association between p-ERK1/2 expression and clinicopathological parameters in 114 patients with breast cancer treated by surgery.

Characteristic	Cases	p-ERK1/2		
		Low(%)	High(%)	P value
**Group**				
NTNBC^a^	69	33(28.9%)	36(31.6%)	
TNBC^b^	45	1(0.9%)	44(38.6%)	<0.001***
**Age**				
<50	73	20(17.5%)	53(46.5%)	
≥50	41	14(12.3%)	27(23.7%)	0.450
**Menopause**				
Pre-menopause	63	21(18.4%)	42(11.4%)	
Post- menopause	51	13(36.8%)	38(33.3%)	0.363
**Tumor size(cm)**				
<3	32	8(7%)	24(21.1%)	
≥3	82	26(22.8%)	56(49.1%)	0.482
**LN^c^ metastasis**				
No	50	17(14.9%)	33(28.9%)	
Yes	64	17(14.9%)	47(41.2%)	0.389
**TNM stage**				
I+II	62	22(19.3%)	43(37.7%)	
III	51	12(10.5%)	37(32.5%)	0.280
**ER status**				
Negative	34	10(8.8%)	24(21.1%)	
Positive	80	50(43.9%)	30(26.3%)	0.001**
**PR status**				
Negative	64	10(8.8%)	54(47.4%)	
Positive	50	24(21.1%)	26(22.8%)	<0.001***
**Her-2 status**				
Negative	70	10(8.8%)	60(52.6%)	
Positive	43	23(20.2%)	20(17.5%)	<0.001***
**Local relapse**				
No	106	33(28.9%)	73(64.0%)	
Yes	8	1(0.9%)	7(6.1%)	0.432
**Distant metastasis**				
No	84	31(27.2%)	53(46.5%)	
Yes	30	3(2.6%)	27(23.7%)	0.006**

a: Non-Triple-negative Breast cancer; b: Triple-negative Breast cancer; c: lymph node.

**Figure 1:**
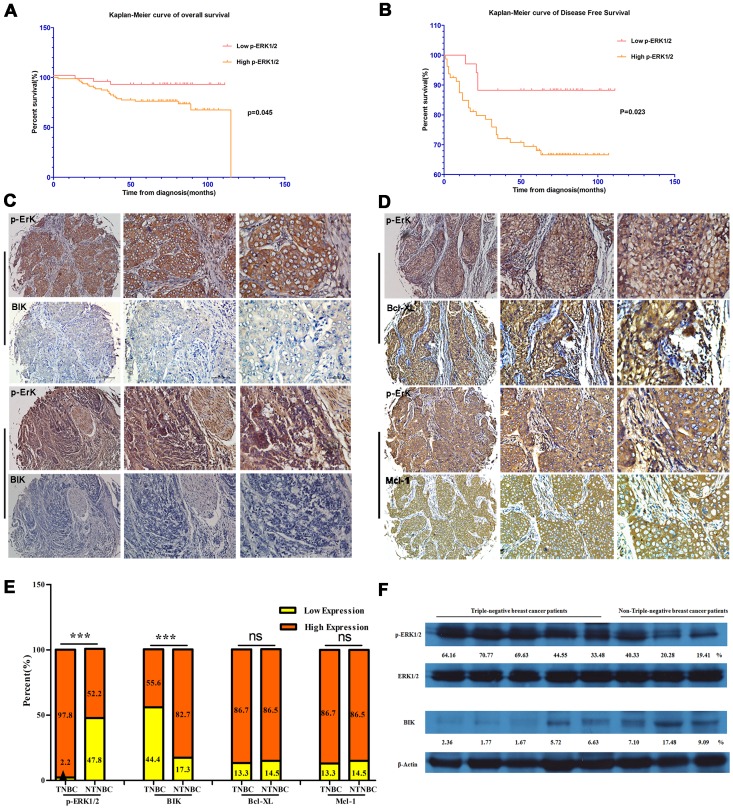
p-ERK1/2 as a meaningful biomarker in triple-negative breast cancer. A and B. Kaplan-Meier curves showed overall survival (OS) and disease-free survival (DFS) according to p-ERK1/2 expression. C. Aberrant p-ERK1/2 expression but absent expression of Bik were exhibited in the same triple-negative breast cancer patients’ samples. D. Co-overexpression of p-ERK1/2 and Bcl-Xl or Mcl-1 in the same breast cancer patients’ samples. E. The distribution of p-ERK1/2, Bik, Bcl-Xl and Mcl-1 status categorized by subgroup. F. The relative expression levels of Bik and p-ERK1/2 in eight breast cancer patients (five TNBC and three NTNBC) by western blotting. Semi-quantitative analysis for Bik and p-ERK1/2 expression: β-actin and total ERK1/2 as control respectively.

Then we proceeded to examine the relationship between p-ERK1/2 expression and patients’ survival. P-ERK1/2 levels in tumors were investigated for overall survival associations using Kaplan-Meier estimates and the log-rank test for significance. Patients with high levels of p-ERK1/2 showed much shorter overall survival time (median overall survival time, 92.2 vs 103.4 months, p = 0.045; Fig 1A) and disease-free survival time (median overall survival time, 78.6 vs 100.3 months, p = 0.023; Fig 1B).

To assess whether p-ERK1/2 was an independent predictor of survival, multivariate COX proportional hazard regression was performed. Multivariate analysis demonstrated elevated p-ERK1/2 was related to decreased disease-free survival (p = 0.045; HR = 2.934; 95%CI 1.022–8.420; Table 2) and can be an independent prognostic indicator of disease-free survival.

**Table 2:** Univariate and multivariate analysis for prognostic value of p-ERK1/2 expression in breast cancer patients.

Characteristic	Overall Survival				Disease-Free Survival			
	Univariate	Multivariate			Univariate	Multivariate		
	P value	P value	HR	95% CI	P value	P value	HR	95% CI
**Age (<50 vs ≥50y)**	0.743	_	_	_	0.474	_	_	_
**Tumor size (<3 vs ≥3 cm)**	0.162	_	_	_	0.214	_	_	_
**LN^a^ infiltrated (No vs Yes)**	0.001**	0.574	1.471	(0.382–5.666)	<0.001***	0.326	1.782	(0.563 – 5.643)
**TNM stage (I+II vs III)**	<0.001***	0.001**	14.43	(2.880–72.328)	<0.001***	0.003**	5.033	(1.715–14.769)
**Hormoreceptors (Neg^b^ vs Pos^c^)**	0.091	_	_	_	0.244	_	_	_
**Her-2 (Neg vs Pos)**	0.486	_	_	_	0.467	_	_	_
**p-ERK1/2 (Low vs High)**	0.046*	0.085	2.910	(0.862–9.822)	0.023*	0.045*	2.934	(1.022–8.420)

a: lymph node; b: negative; c: positive.

### Aberrant expression of p-ERK1/2 correlated with loss of Bik and overexpression of Bcl-Xl and Mcl-1

To explore the significance of aberrant expression of p-ERK1/2 in TNBC for cancer targeted gene therapy of BikDD, we investigated the correlation between p-ERK1/2 expression and expression of Bik, Bcl-2, Bcl-Xl and Mcl-1 in 114 breast cancer patient samples. We found expression levels of p-ERK1/2 correlated inversely with Bik levels and positively with Bcl-Xl and Mcl-1 levels (p = 0.028, p = 0.004, p = 0.019, respectively; Table 3). As showed in Fig 1C, aberrant p-ERK1/2 expression but absent expression of Bik were exhibited in the same triple-negative breast cancer patients’ samples. Co-overexpression of p-ERK1/2 and Bcl-Xl or Mcl-1 was also observed (Fig 1D). However, there was no significant correlation between Bik levels and Bcl-2 levels (p = 0.273; Table 3). As described above, p-ERK1/2 was particularly over expressed in triple-negative breast cancer. We analyzed the difference in distribution of Bik, Bcl-Xl or Mcl-1 status between TNBC and NTNBC patients. P-ERK1/2 showed high expression in 98% TNBC patients. However, compared with NTNBC patients, Bik tended to show low expression in TNBC (p<0.001; Fig 1E). However, no significant difference was observed in distribution of Bcl-Xl or Mcl-1 status between TNBC and NTNBC patients (Fig 1E). Furthermore, the relative expression levels of Bik and p-ERK1/2 was analyzed in eight patients (five TNBC and three NTNBC) by western blotting. High p-ERK1/2 but low Bik levels were observed in TNBC patients (Fig 1F).

**Table 3:** High levels of p-ERK1/2 correlated with loss of Bik and overexpression of Bcl-Xl and Mcl-1.

	p-ERK1/2			
	Low(%)	High(%)	Total	P value
**Bik**				
Low expression	6(5.3%)	31(27.2%)	37	
High expression	28(24.6%)	49(43.0%)	77	0.028*
**Bcl-2**				
Low expression	10(8.8%)	24(21.1%)	34	
High expression	16(14.0%)	64(56.1%)	80	**0.273**
**Bcl-Xl**				
Low expression	10(8.8%)	24(21.1%)	34	
High expression	6(5.3%)	74(64.9%)	80	0.004**
**Mcl-1**				
Low expression	9(7.9%)	25(21.9%)	34	
High expression	7(6.1%)	73(64.0%)	80	0.019*

It has been reported p-ERK1/2 triggers ubiquitin-dependent degradation of Bik by phosphorylating Bik on Ser^124^ [12], and mediates up-regulation of expressions of Bcl-2, Bcl-Xl and Mcl-1 [23]. So these data implied loss of Bik but overexpression of anti-apoptotic Bcl-2 homologs mediated by aberrant p-ERK1/2 expression can be a notable genomic defect of TNBC and an important mechanism by which TNBC cells evade apoptosis. Therefore, inducing apoptosis by introducing BikDD into TNBC cells could be a promising therapeutic approach.

### BikDDA, a novel therapeutic mutant, had a longer half-life than BikDD

What’s more, we came up with a new idea: on the basis of BikDD, we generated a novel mutant, BikDDA, in which Serine 124, the conserved phosphorylation site of p-ERK1/2, was changed to Alanine, the one unphosphorylable for p-ERK1/2, to prevent subsequent ubiquitin-proteasome degradation.

**Figure 2:**
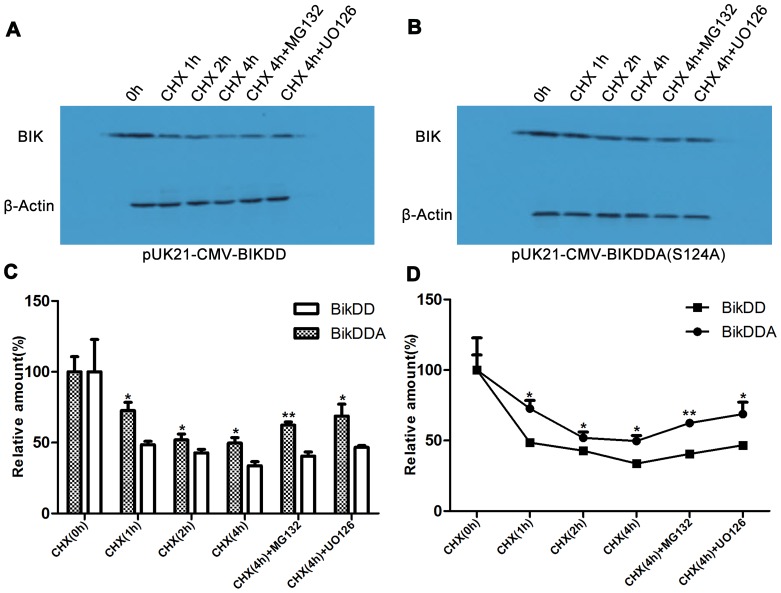
BikDDA had a longer half-life than BikDD. A. 293 cells were transfected for 16h with same amount of pUK21- BikDD or pUK21-BikDDA and treated with protein biosynthesis inhibitor cycloheximide (CHX), proteasome inhibitor MG132 or Mek1/2 inhibitor UO126. B. Histogram and line charts showed the gray scale quantitative analysis for western blotting using Gel-pro software. The relative levels before CHX treatment were set as 100%. These results demonstrated that BikDDA degradation rate was lower than BikDD.

To test whether BikDDA had a longer half-life than BikDD, we used western blotting to detect their relative protein degradation rates. After inhibiting protein biosynthesis by cycloheximide, we detected BikDD and BikDDA at the points 0, 2 and 4h (Fig 2A). The graph intuitively showed that compared with BikDD,BikDDA degraded more slowly(Fig 2B). Besides, the MEK1/2/5 inhibitor UO126, or the proteasome inhibitor MG132 abolished the difference (Fig 2A). These data demonstrated that after S124A mutation BikDDA showed increased stability.

### BikDDA eliminated TNBC cells more powerfully than BikDD

**Figure 3:**
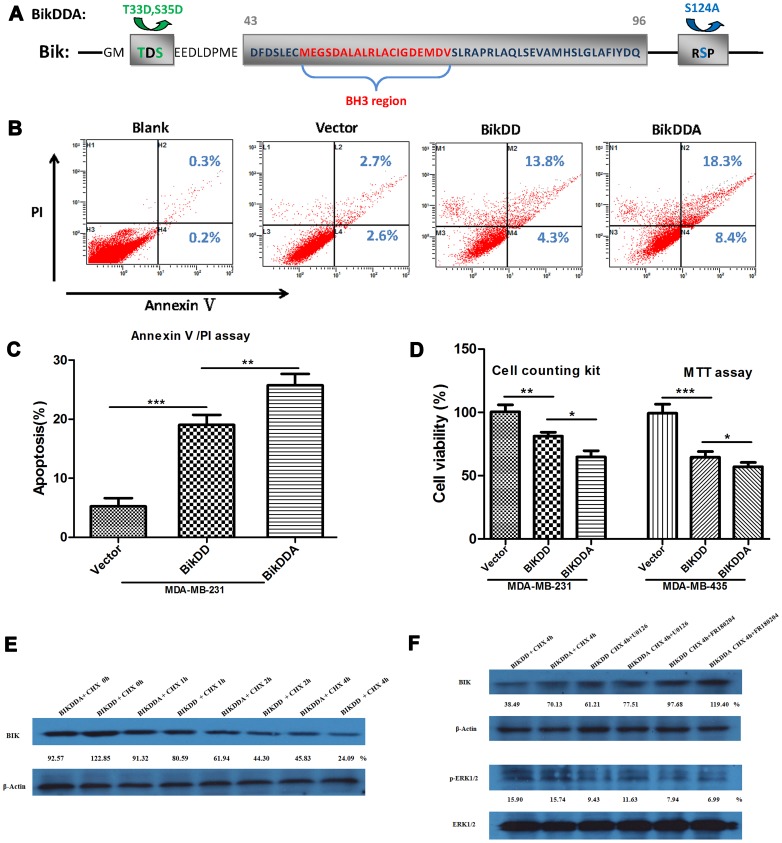
BikDDA eliminated TNBC cells more powerfully than BikDD. A. S124A and previous mutant sites T33D and S35D didn’t locate in the pro-apoptotic region of Bik. B. MDA-MB-231 cells were transfected for 28h with the same amount of pUK21, pUK21-BikDD or pUK21-BikDDA. Apoptotic cells were monitored by Annexin V/PI staining and flow-cytometry analysis. The right-lower or right upper quadrant of each plot showed early apoptotic or late apoptotic cells. C. Histogram directly showed the enhanced apoptosis-inducing activity of BikDDA. D. The cytotoxicity of BikDD and BikDDA were analyzed by Cell counting kit and MTT assay (setting at 100% in vector group). E. Western blotting was performed to confirm the prolonged half-life of BikDDA in 231 cells. F. MEK1/2 inhibitor UO126 and selective ERK1/2 inhibitor FR180204 increased the stability of BikDD in 231 cells.

Next we focused on the apoptotic activity of BikDDA. Out of question, the core BH3 domain is crucial for pro-apoptotic activity. Furthermore, the amino acids 43-96 region of Bik, encompassing the BH3 domain, is essential for efficient heterodimerization with Bcl-2 homology to induce apoptosis [24]. Therefore, to avoid defects caused by mutant in eliciting cell death, the mutant sites couldn’t lie in this region. So we mutated Ser^124^ which was outside the region rather than Lys^61^ on which amino site p-ERK1/2 interacts with Bik but locates in the pro-apoptotic region. Besides, previous mutants T33D and T35D were also outside the region (Fig 3A).

To investigate whether BikDDA was more potent than BikDD, AnnexinV/PI assay was performed. After transfection into MDA-MB-231, both BikDD and BikDDA induced significant cell apoptosis; moreover BikDDA exerted stronger apoptosis-inducing effects (Fig 3B). The graphs directly showed the greater pro-apoptotic potency of BikDDA (Fig 3C). Besides MTT assay and cell counting kit approved the enhanced cytotoxicity of BikDDA to TNBC cells (Fig 3D). Then we confirmed the prolonged half-life of BikDDA via western blotting analysis in MDA-MB-231 cell line (Fig 3E). Fig 3F showed that the MEK1/2 inhibitor UO126 and selective ERK1/2 inhibitor FR180204 can increase the stability of BikDD. Taken together, BikDDA, with a longer half-life, could eliminate TNBC cells more powerfully than BikDD.

### P-ERK1/2 was associated with the response to adjuvant 5-FU based chemotherapy in breast cancer patients and BIKDDA can enhance the therapeutic effect of 5-FU in vitro

**Figure 4:**
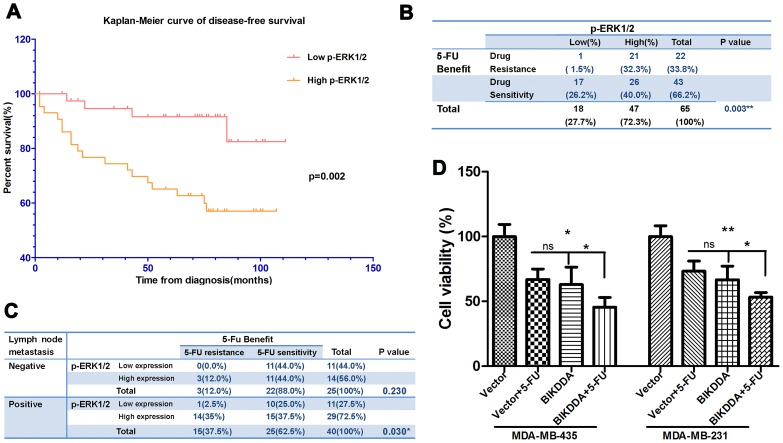
Higher p-ERK1/2 correlated with 5-FU resistance and BIKDDA could enhance the cytotoxicity of 5-FU. A. Kaplan-Meier curve of disease-free survival showed the decreased disease-free survival time of high p-ERK1/2 group who received adjuvant 5-FU based chemotherapy than the low. B and C. High levels of p-ERK1/2 correlated with 5-FU resistance (patients with metastasis and/or recurrence after adjuvant 5-FU based chemotherapy). After stratified by lymph node metastasis the correlation also exhibited statistics significance in metastasis positive cohort. D. 435 and 231 cells were transfected with vector control or BikDDA in combination with 25 ug/ml 5-FU or not, 24h later cell viability was analyzed by MTT assay (vector transfected only setting as 100%). These data showed BIKDDA can enhance the cytotoxicity of 5-FU.

It has been reported high levels of p-ERK1/2 correlated with chemotherapy [25], [26], To analyze the relationship of p-ERK1/2 protein expression with response to adjuvant 5-FU based chemotherapy, patients who received adjuvant 5-FU based chemotherapy after surgery were selected (n = 65) and two test models were used. In model I wherein treatment response was based on disease-free survival, patients with high p-ERK1/2 levels had decreased disease-free survival than those with low levels (median, 74.5 vs 102.5 months; log-Rank, p = 0.014; Fig 4A).

In model II, patients (n = 65) were dichotomized: after adjuvant 5-FU based chemotherapy, patients with metastasis and/or recurrence were regarded as 5-FU resistance group and otherwise as 5-FU sensitivity group. χ^2^ square test showed that high levels of p-ERK1/2 correlated with 5-FU resistance (32.3% vs 1.5%, p = 0.003; Fig 4B). What’s more, after stratified by lymph node metastasis, the correlation also exhibited statistical significance in metastasis positive cohort (35% vs 2.5%, p = 0.030; Fig 4C).

Furthermore, we explored whether BikDDA can enhance the anti-tumor effects of 5-FU. MTT assays showed the combination of 5-FU and BikDDA increased the therapeutic efficacy in MDA-MB-435 and MDA-MB-231 cells (Fig 4D).

## Discussion

In this study, we found aberrant expression of p-ERK1/2 was a meaningful molecular phenotype in TNBC, not just for its prognosis value, but also a guideline for cancer targeted gene therapy of BikDD. As reported at the molecular levels p-ERK1/2 accelerates Bik degradation and promotes anti-apoptotic Bcl-2 homolog expressions [12], [23], in this study we discovered p-ERK1/2 expression inversely correlated with Bik and positively with Bcl-Xl and Mcl-1 in breast cancer patients’ tissues. In addition, it has been reported activation of ERK1/2 can also decrease Bim-EL mRNA, another pro-apoptotic member of Bcl-2 family, promotes Bim-EL rapidly dissociating from MCL-1 and Bcl-Xl and accelerates its degradation [27], [28]. So these results allow us to get a deeper insight into the malignancies generated by over-activated ERK1/2: p-ERK1/2 inhibits the mitochondrial pathway of apoptosis to evade cell death by discriminating in favor of anti-apoptotic Bcl-2 homologs.

It has been demonstrated that except the interaction with anti-apoptotic Bcl-2 homolog, the proapoptotic function of Bik also stems from its ability to block nuclear translocation of p-ERK1/2 [29]. Since highly activated ERK1/2 was a notable defect contributing to evasion of apoptosis, introducing the pro-apoptotic gene Bik into TNBC cells to repair the defect was an excellent therapeutic strategy. However, due to high levels of p-ERK1/2, BikDD degraded faster in TNBC cells. So we generated BikDDA based on BikDD, a novel mutant form of Bik, as a therapeutic gene for TNBC. We demonstrated the longer half-life and more potent apoptosis-inducing activity of BikDDA in TNBC cells. So BikDDA can be a novel therapeutic gene in triple-negative breast cancer targeted gene therapy.

5-FU is a main chemotherapeutic drug for TNBC. However resistance to 5-FU acquired by some cancer phenotypes remains significant limitation to its use. Deeply understanding the molecular characterization of TNBC can lead to new therapeutic strategies. In this study we showed high levels of p-ERK1/2 contributed to the resistance of adjuvant 5-FU based chemotherapy in breast cancer patients. As all roads to successful eradication of cancer cells by chemotherapy exploit the induction of apoptosis [30]. Loss or non-function of pro-apoptotic members of Bcl-2 family and overexpression of anti-apoptotic Bcl-2 homology mediated by p-ERK1/2 may be a vital mechanism of 5-FU resistance. So introducing pro-apoptotic BikDDA is a promising therapeutic strategy to enhance the anti-tumor effects of 5-FU.

## Supporting Information

Figure S1 **Representative images for immunohistochemical staining intensity (0-negative, 1-weak, 2-moderate, and 3-strong).**
(TIF)
