# Supplementary figures and images for: BikDDA, a Mutant of Bik with Longer Half-Life Expression Protein, Can Be a Novel Therapeutic Gene for Triple-Negative Breast Cancer
Source: PLoS One. 2014 Mar 17;9(3):e92172. doi: 10.1371/journal.pone.0092172 (PMC3956915; doi:10.1371/journal.pone.0092172)

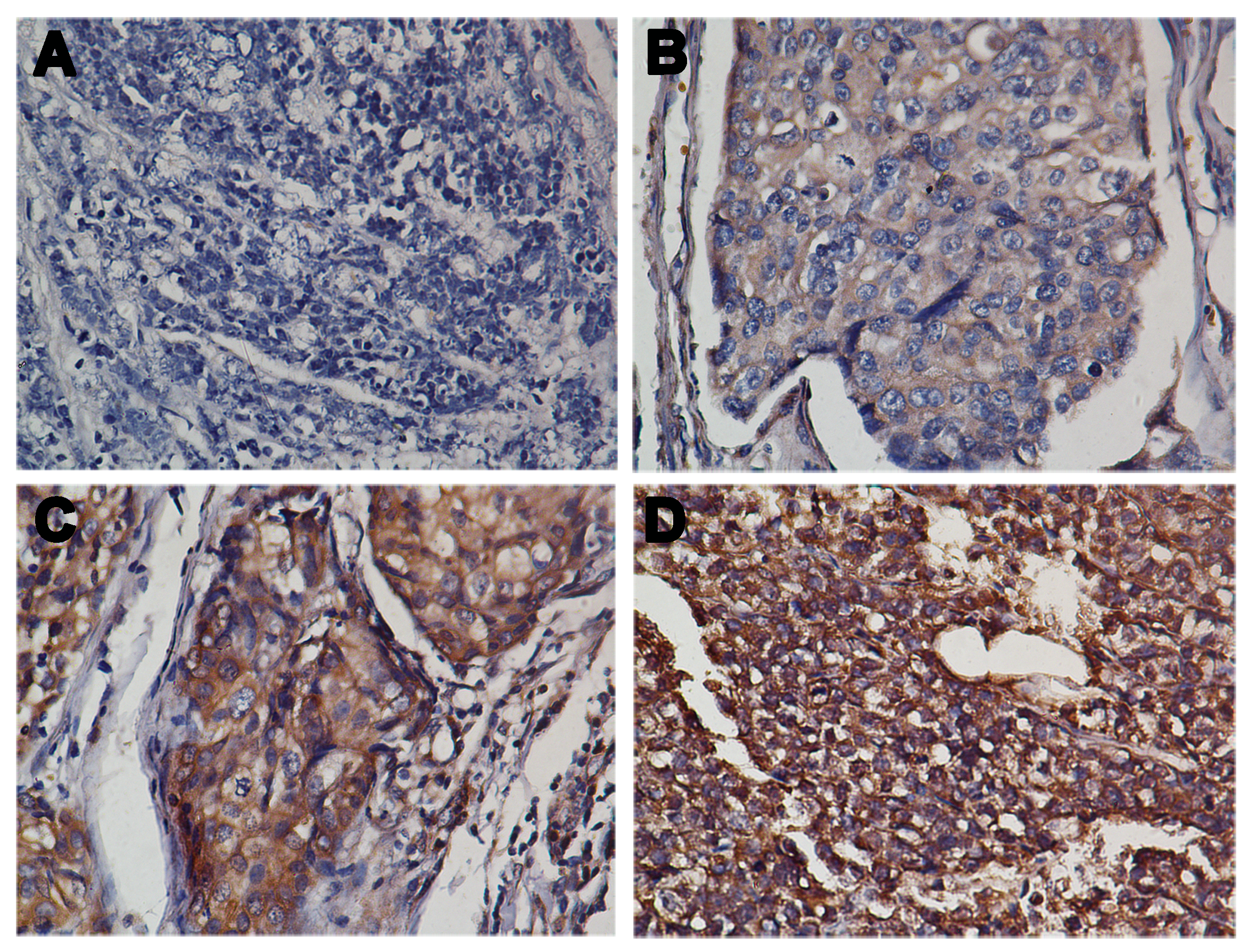

Supplement: Figure S1 — Representative images for immunohistochemical staining intensity (0-negative, 1-weak, 2-moderate, and 3-strong). (TIF) [file pone.0092172.s001.tif]
